# Coevality of Systemic Lupus Erythematosus With Sickle Cell Trait: A Not So Uncommon Entity

**DOI:** 10.7759/cureus.10119

**Published:** 2020-08-29

**Authors:** Dhriti Sundar Das, Debananda Sahoo

**Affiliations:** 1 General Medicine, All India Institute of Medical Sciences, Bhubaneswar, IND; 2 Internal Medicine, All India Institute of Medical Sciences, Bhubaneswar, IND

**Keywords:** sickle cell trait, systemic lupus erythematosus, lupus pneumonitis

## Abstract

The coexistence of systemic lupus erythematosus (SLE) with sickle cell trait is quite sparingly reported in literature. Here, we narrate the case of a 17-year-old girl from Eastern India with sickle cell trait who presented with acute lupus pneumonitis. The challenges to the final diagnosis of SLE with sickle cell trait were because of the often lesser degree of clinical suspicion at the outset. In this report, we discuss this not so uncommon combination of conditions and review related literature. This girl, who was a known case of sickle cell trait, presented with fever, cough, shortness of breath with subsequent rashes, oral ulceration, high erythrocyte sedimentation rate (ESR) and proteinuria. After ruling out infective causes, she was found to be antinuclear antibody (ANA) positive and with stage 4 lupus nephritis. Emphasis should be given to the presence of autoimmune conditions in patients with sickle hemoglobinopathies, including sickle cell trait wherein atypical or systemic involvement may occur. Such association holds more importance as sickle hemoglobinopathies is one of the major hemoglobinopathies reported in this part of the country.

## Introduction

Sickle hemoglobinopathies, a group of commonly encountered genetic disorders that include sickle cell trait, sickle cell disease, and sickle Beta−Thalassemia, are prevalent in this eastern part of India [1,2]. Due to the multisystem involvement and manifestations, the diagnosis of lupus in patients with hemoglobinopathies may often get delayed [3,4]. We report a case of known sickle cell trait whose clinical features and subsequent presentations lead to the diagnosis of lupus.

## Case presentation

**Figure 1:**
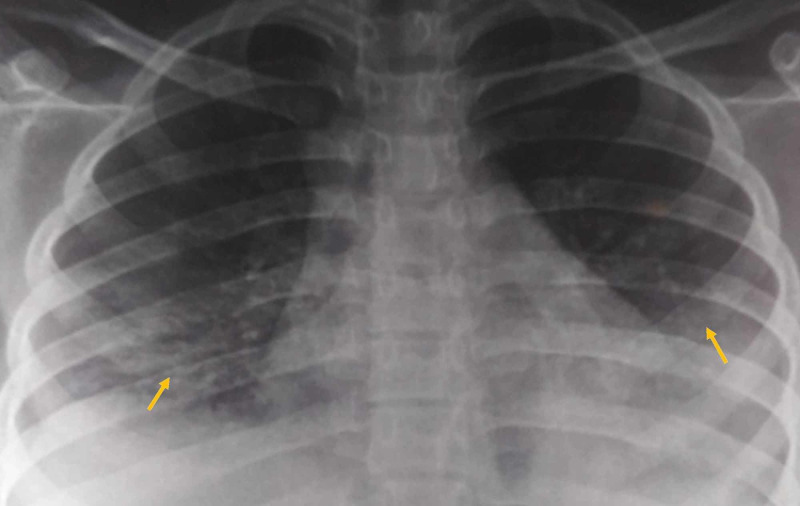
Chest x-ray posteroanterior (PA) view showing bilateral patchy infiltrates

**Figure 2:**
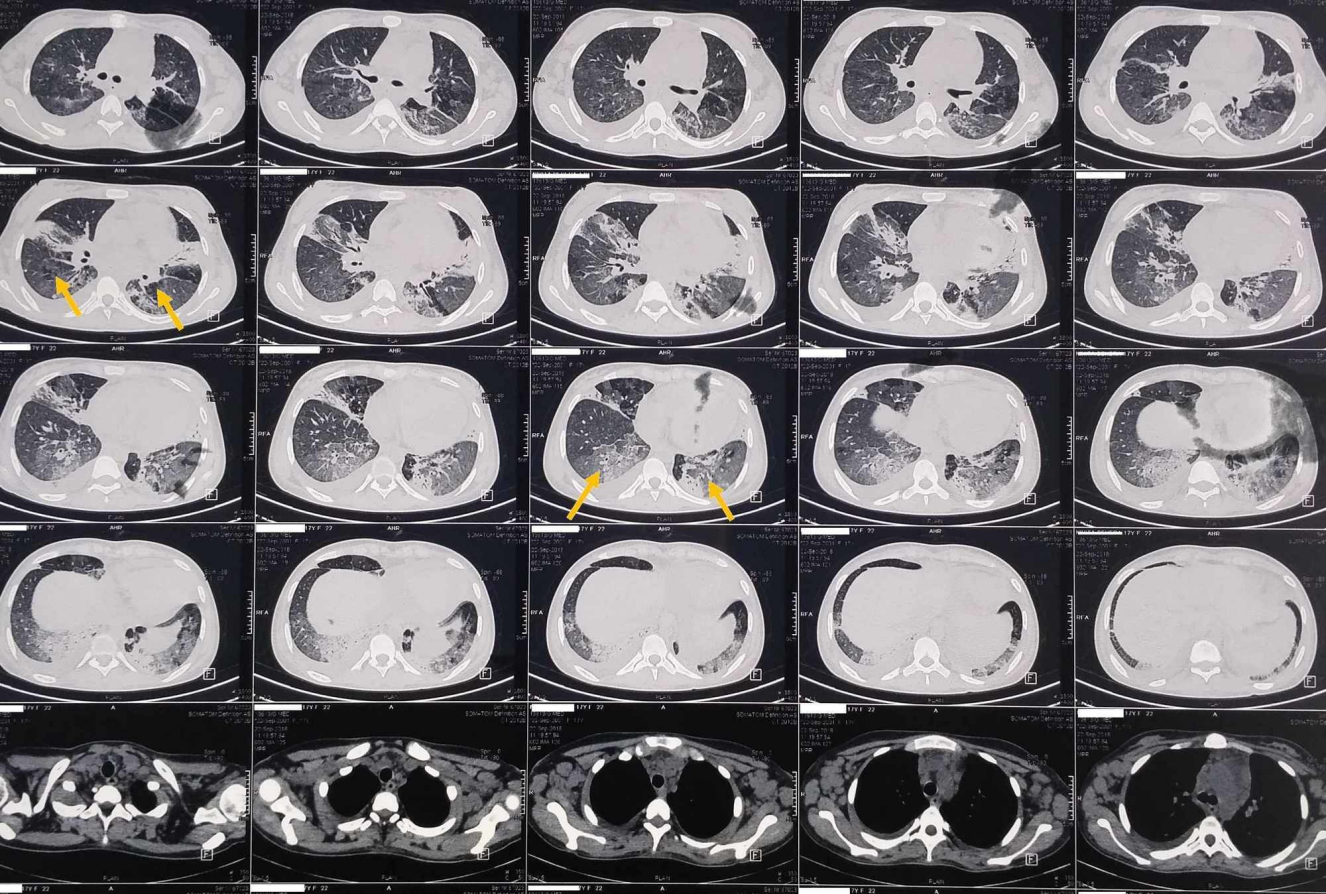
CT thorax showing bilateral ground-glass opacities

A 17-year-old girl presented with low-grade fever, not associated with chills and rigor, cough with expectoration for two weeks, non-radiating chest pain, and breathlessness of three days duration. She was a known case of sickle cell trait. The cough was associated with hemoptysis. She had no history of joint pain or photosensitivity rashes. Past history revealed cervical lymphadenopathy a few months back with biopsy showing reactive lymphadenitis; apart from this, no other past similar illness was reported. Family history was not suggestive. She had no history of tuberculosis or any contact history of tuberculosis. On examination, pulse was 112/min, blood pressure 136/86 mmHg, oxygen saturation being 90% on room air and respiratory rate being 30 per minute. On auscultation, chest revealed bilateral crepitations and wheezes. Cardiac and neurological examinations were unremarkable. Subsequently, her saturation dropped to 84% on room air. She was started on IV antibiotics, nebulization with salbutamol, isotonic normal saline. Further evaluation revealed high erythrocyte sedimentation rate (ESR; 151 mm at the end of the first hour) with anemia, rashes over both feet, bilateral cervical lymphadenopathy, painless oral ulcer and amenorrhea. Her initial total leucocyte counts and renal function test were normal. There was mild thrombocytopenia (1.26 lakh per cumm) and hypoproteinemia with hypoalbuminemia. Routine urinalysis showed 3+ proteinuria. Chest x-ray showed bilateral patchy infiltrates (Figure 1). Peripheral blood smear showed features of hemolytic anemia with target cells. Direct Coombs test was positive. Serum ferritin was high. The test for antinuclear antibodies (ANA) was positive (3+; mixed pattern), and dsDNA was also positive, fulfilling the American College of Rheumatology criteria for the diagnosis of systemic lupus erythematosus (SLE). Ultrasound of the abdomen showed only splenomegaly. Computed tomography (CT) scan of thorax revealed bilateral ground-glass opacities with right pleural effusion with bilateral mediastinal, axillary lymphadenopathy (Figure 2). Sputum culture showed no growth. Sputum was negative for acid-fast bacilli (AFB) and cartridge-based nucleic acid amplification test (CBNAAT). Twenty-four-hour urine protein was 3521 mg. Echocardiography was normal. Perinuclear antineutrophil cytoplasmic antibodies (P-ANCA) and cytoplasmic antineutrophil cytoplasmic antibodies (C-ANCA) were negative. The patient responded with pulse methylprednisolone and then converted to oral steroids and started on hydroxychloroquine. The patient was discharged to follow up with renal biopsy report, which revealed proliferative and sclerosing lesions involving more than 50 percent of glomeruli suggestive of stage 4 lupus nephritis. However, the red blood cells (RBCs) within the vessels did not show evidence of sickling. 

## Discussion

The present case of sickle cell trait (SCT) and SLE highlights the importance of diagnosing such association with utmost clinical suspicion. The diagnosis of SCT is usually made long before SLE is evident [5]. The coexistence of these diseases is of great interest, but the paucity of cases that are being reported from this part of the country implies it is uncommon. Moreover, since sickle hemoglobinopathies are the major hemoglobinopathies in this eastern state of India (Odisha), therefore such association holds more importance and relevance from clinical and management point of view of such cases in this part of the country [1]. With the overlapping clinical features and reporting of ANA positivity in patients with sickle cell disease (SCD), diagnosing lupus is indeed very challenging to the clinicians at large [3,5]. However, very few cases of lupus are reported with the SCT variant of hemoglobinopathies [4]. Patients with SCD have an abnormal alternate pathway of the complement system and this may make them prone to the development of autoimmune diseases [3,6]. The array of symptoms of both the conditions may, at times, be confusing to substantially delineate out the primary disease and therefore, one should have a high index of clinical suspicion on encountering such symptomatology to make an early diagnosis. The high titers of ANA positivity in sickle hemoglobinopathies especially taxes physicians in current clinical practice.

Large clinico-epidemiological studies are indeed required to delve more into such association of immune disorders in patients with sickle hemoglobinopathies.

## Conclusions

This report highlights the importance of having a high degree of clinical suspicion in diagnosing such cases at the earliest possible time and starting treatment without any further delay to prevent complications. Such association holds more relevance in our setting as sickle hemoglobinopathies are the commonest hemoglobinopathies in this eastern state of India, i.e., Odisha.
